# Workplace Cold and Perceived Work Ability: Paradoxically Greater Disadvantage for More vs. Less-Educated Poultry Industry Workers in Thailand

**DOI:** 10.3389/fpubh.2021.762533

**Published:** 2021-12-01

**Authors:** Wantanee Phanprasit, Pajaree Konthonbut, Wisanti Laohaudomchok, Chaiyanun Tangtong, Tiina M. Ikäheimo, Jouni J. K. Jaakkola, Simo Näyhä

**Affiliations:** ^1^Department of Occupational Health, Faculty of Public Health, Mahidol University, Bangkok, Thailand; ^2^Center for Environmental and Respiratory Health Research, University of Oulu, Oulu, Finland; ^3^Department of Community Medicine, University of Tromsø, Tromsø, Norway

**Keywords:** occupational, cold exposure, work ability, education, poultry industry, Thailand

## Abstract

The association between worksite temperature and perceived work ability (WA) in various educational classes remains unknown. Therefore, we interviewed 286 poultry industry workers in Thailand about their WA and linked their responses to worksite temperature. WA was based on the self-assessment of current work ability compared with their lifetime best ability (scores 0–10). Education was classified as high (university or vocational school) or low (less education). Temperature was classified as cold (−22–10°C) or warm (10–23°C). WA and the occurrence of a low WA were regressed on worksite temperature, education, and their interaction with the adjustment for sex, age, job category, physical work strain, moving between cold and warm sites, thermal insulation of clothing, relative humidity, and air velocity. The average worksite temperature was 10°C for high- and 1°C for low-educated workers. The average WA score was 8.32 (SD, 1.33; range, 4–10) and classified as low (<8) in 23% of the workers. In highly-educated workers, the adjusted mean WA decreased from 9.11 in the warm areas to 8.02 in the cold areas and the prevalence of a low WA increased from 11 to 30%, while no significant change was observed in less-educated workers. The WA score was estimated to decline by 10% more (95% CI, 4–16%) in the cold areas for the more vs. less-educated workers and the prevalence of a poor WA was estimated to increase 3.09 times (95% CI, 1.43–5.45) more. Highly-educated workers in this industry are a risk group that should be given customized advice.

## Introduction

Food industry workers often work at temperatures down to −20°C and commonly suffer from various cold-related harms. For example, in Thailand, 82% of poultry industry workers report degradation of performance that they attribute to workplace cold (1). The means used to prevent or mitigate cold harm include protective clothing (2) and restricting the time spent in cold. Despite these measures, the prevalence of various cold adversities remains high and most workers in this industry suffer from at least some cold-related symptoms or degradation of performance (1–4). The vulnerability of workers in the food and beverage industry in this country is also shown by the high number of work injuries (5). Since Thailand is the 8th largest provider of chicken meat and products worldwide (6) and employs a large proportion of the overall labor force, any effective preventive efforts could produce large reductions in cold-related illness burden and economic losses.

One strategy to reduce the cold-related burden at workplaces would be to search for vulnerable subgroups of workers and develop intensified measures to protect them. A number of studies have reported the breakdown of workers according to various personal or workplace-related vulnerability factors; however, they used them only for sample descriptions or to adjust for confounding factors (4, 7–9). Our previous studies of poultry industry workers in Thailand identified highly-educated workers as a risk group for cold adversity because a disproportionately large proportion of them reported various cold symptoms, especially degradation of performance (1, 2). This was unexpected, since in general populations, a high education level is associated with high social class and better health as shown by a wide variety of health indicators (10, 11).

Since previous studies on this topic have focused on individual symptoms and performance items that are caused by cold according to the own assessment of the worker, we evaluated whether actual measured temperature at the workplace is associated with perceived work ability (WA), which we used as a summary indicator for subjective work-related health and functional ability. In particular, we test the hypothesis that the association of WA with workplace cold is stronger among more vs. less-educated workers.

## Materials and Methods

### Study Population

**Figure 1:**
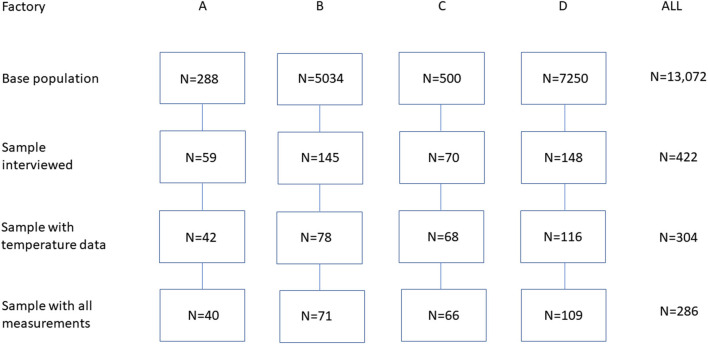
Flowchart of the participant sampling scheme.

The database used was previously described in detail (1–3). The sample was drawn from four chicken meat factories in Central and Northeastern Thailand (Figure 1). All the workers of these factories (288, 5,034, 500, and 7,250 people, respectively) were invited to participate in a health survey to determine the prevalence of cold symptoms and other occupational hazards. Power calculations indicated that a sample of 420 workers was adequate for this purpose (1). Of the 13,072 workers in the factories in question, 59, 145, 70, and 148 people, respectively (*n* = 422) were selected by convenience sampling and interviewed. The final number of subjects in each factory was determined in relation to their availability during regular working hours and the work schedules of the study team. Worksite temperature, relative humidity, and air velocity were measured in cold storages and manufacturing halls and offices in which workers were willing to participate. Temperature was available for 304 workers; of them, 286 workers for whom data for all the relevant personal and workplace factors were available were included in the present analysis. The work in the factories consisted of chicken meat cutting, processing, storing, packing, and paperwork. The work was performed in cold storages, manufacturing halls, and offices.

The study plan was approved by the Ethical Review Committee for Human Research, Faculty of Public Health, Mahidol University, Bangkok, Thailand (no. MUPH 2017-198). The interviewees were informed that their participation was strictly voluntary and that all the information would remain confidential. All the participants provided a written informed consent.

### Interview

The participants were interviewed by trained interviewers to collect their personal details, living habits, work-related factors, and cold-related symptoms by using a structured questionnaire (1). Perceived WA was elicited by the following question: “If your best WA during your lifetime received a score of 10, what score would you assign to your present WA?” The question was selected from a series of seven questions that constitute the Finnish Work Ability Index, which has been widely used in occupational research (12–15). Lundin et al. (13) measured the predictive ability of this single question in terms of sickness absence during a follow-up period of 4 years. Their receiver operating characteristic analysis yielded an area under the curve value of 0.78 during the 1st year of follow-up and 0.72 during all the 4 years, indicating acceptable validity. The respondents were also asked to indicate their highest attained educational level (university, vocational school, high school, middle school, primary school, or less). In the data analysis, education was dichotomized as high (university or vocational school) or low (all the other levels). Sensitivity analyses were also performed by using a stricter definition of high education (university vs. all the other educational levels). Other questions asked about job category (office work; other work, including manufacturing, cold storage work, or forklift driving), physical strain at work (light: sedentary or other light work; heavy: medium heavy or heavy work), body weight and height [converted to body mass index (**BMI**) (kg/m^2^)], smoking (current smoker; others: never smoked, previously smoked**)**, and alcohol consumption (weekly; other: occasionally, monthly, never). The respondents were also asked what temperature they considered cold, how many times a day they moved between cold and warm sites, how many hours they spent at temperatures below zero, and leisure time physical exercise (times/week). The interviewees were also asked to indicate the clothing items used at work (28 separate items) (16). The thermal insulation of the clothing ensemble (clo) was calculated as the basic thermal insulation I_cl_ = 0.161 + 0.835 ∑ I_clu_, where I_clu_ denotes the clo value of each clothing item.

### Measurements

Air temperature and relative humidity were measured by using a 303 C thermo-hygrometer (Shenzhen Graigar Technology Corporation Ltd., Shenzhen, China) and air velocity by using the VelociCalc® 9545 (TSI Incorporated, Minnesota, USA). The measurements were conducted at several points in the regular work areas of the participants. The regular work area was assessed by the question: “Where do you work most of the time?” The area could be a cold storage, a manufacturing hall, or an office; measurements were taken at 20, 13, and four sites, respectively (*n* = 37). In areas where the temperature and air velocity varied, the minimum and maximum values were recorded and expressed as averages. The values recorded in each working area were linked to the study participants of the same area.

### Data Analysis

The educational classes were first compared with respect to personal and workplace factors by using the one-way ANOVA and the chi-squared test for heterogeneity. Ordinary linear regressions were conducted with the WA score as the outcome variate and worksite temperature (cold, −22 to 9.9°C; warm, 10–23°C) (17) and education as the explanatory factors. Next, a low WA (<8, coded as 1, 0) was regressed on temperature and education by using a generalized linear model with the logit link and quasibinomial error distribution. Adjustments were made for sex, age, job category, physical work strain, moving between cold and warm sites, thermal insulation of clothing (clo), relative humidity, and air velocity. The interaction between educational class and temperature was entered into the models to determine how the effect of the temperature was modified by education. Additional variables included smoking, alcohol consumption, leisure-time physical activity, BMI, and hours spent at <0°C, but they did not affect the parameter estimates and were omitted. The results are expressed as model-based adjusted mean WA and the prevalence of a low WA calculated as marginal means (18) that were converted to ratios of means and prevalence ratios together with their 95% CIs. The results were obtained by using R software release 3.50 (https://cran.r-project.org/). All the regressions were adjusted for stratified sampling by using the svydesign function of R.

## Results

### Overall Characteristics of the Participants

Of the 286 workers studied, 166 (58%) were men and 120 (42%) were women. The average age was 32.7 years (SD, 10.2; range, 18–57 years). A total of 257 participants (90%) were manufacturing workers (48%), storage workers (31%), or forklift drivers (11%), while 29 (10%) were office staff. Women were more likely to work in the office setting (72 vs. 28%); office workers were more likely to be highly educated (97 vs. 18%), but the mean age differed little between the office and other work settings (33.4 vs. 30.7 years, respectively). Among those included, 58**%** performed heavy work **(**men, 78**%**; women, 29**%)**. The mean BMI was 24.1 kg/m^2^ (SD, 4.7 kg/m^2^; range, 15.6–27.5 kg/m^2^); 36% were classified as obese (BMI > 25 kg/m^2^) (19). A total of 33% of the participants were smokers, 15% of the participants consumed alcohol on a weekly basis, and 43% of the participants exercised in their leisure time at least once a week. A total of 12 respondents (4%) reported having elevated blood pressure as diagnosed by a doctor, while <5% of respondents reported other diagnosed medical conditions.

### More vs. Less-Educated Workers

#### Personal Characteristics

Table 1 compares the personal and workplace characteristics of the more vs. less-educated workers. A total of 75 participants (26%) had a high educational level (university, 37; vocational school, 38). A higher proportion of the more educated workers were women (57 vs. 43%; *p* ~ 0.003). Most office workers (97%) had a high educational level, but among all of the highly-educated workers, only 37% worked in offices, while the remaining 63% were manufacturing or storage workers or forklift drivers. Other differences between the more and less-educated workers were that the former group featured fewer workers who engaged in heavy work (40 vs. 64%; p ~ 0.001), had a higher BMI (25.1 vs. 23.7 kg/m^2^; *p* ~ 0.024), or were smokers (12 vs. 41%; *p* <0.001).

**Table 1:** Percentages (numbers) of participants according to personal characteristics and workplace environmental conditions: high- and low-educated workers compared.

	**Educational class^a^**		**All**
	**High**	**Low**	
**Sex**
Men	42.7 (32)	63.5 (134)	58.0 (166)
Women	57.3 (43)	36.5 (77)	42.0 (120)
p ~^b^		0.003	
**Age (years)**
13 to 29	50.7 (38)	44.5 (94)	46.2 (132)
30 to 57	49.3 (37)	55.5 (117)	53.8 (154)
*p* ~^b^		0.437	
**Obesity**
Obese (BMI ≥ 25.0 kg/m^2^)	42.7 (32)	34.1 (72)	36.4 (104)
Normal (BMI <25.0 kg/m^2^)	57.3 (43)	65.9 (139)	63.6 (182)
*p* ~^b^		0.237	
**Job category**
Office work	37.3 (28)	0.5 (1)	10.1 (29)
Other^c^	62.7 (47)	99.5 (210)	89.9 (257)
*p*<^b^		0.001	
**Heavy work^d^**
Light	60.0 (45)	36.0 (76)	42.3 (121)
Heavy	40.0 (30)	64.0 (135)	57.7 (165)
*p* ~^b^		0.001	
**Leisure time exercise (times/week)**			
0	50.7 (38)	58.8 (124)	56.6 (162)
1+	49.3 (37)	41.2 (87)	43.4 (124)
p ~^b^		0.280	
**Smoking**
Smoker	12.0 (9)	40.8 (86)	33.2 (95)
Non-smoker	88.0 (66)	59.2 (125)	66.8 (191)
*p*<^b^		0.001	
**Alcohol consumption**
Weekly	12.0 (9)	16.6 (35)	15.4 (44)
Less often	88.0 (66)	82.0 173)	83.6 (239)
Unknown	0.0 (0)	1.4 (3)	1.0 (3)
p ~^b^		0.422	
**Worksite temperature**
Cold (<10°C)	44.0 (33)	72.5 (153)	65.0 (186)
Warm (≥10°C)	56.0 (42)	27.5 (58)	35.0 (100)
*p*<^b^		0.001	
**Relative humidity (%)**
Below median (27.0 to 40.5)	38.7 (29)	55.0 (116)	50.7 (145)
Above median (40.6 to 72.0)	61.3 (46)	45.0 (95)	49.3 (141)
*p* ~^b^		0.022	
**Air velocity (m/s)**
Below median (0.0 to 0.38)	33.3 (25)	55.9 (118)	50.0 (143)
Above median (0.39 to 3.00)	66.7 (50)	44.1 (93)	50.0 (143)
*p*<^b^		0.001	
**Moving between cold and warm sites**			
0 to 3 times/day	24.0 (18)	14.7 (31)	17.1 (49)
≥ 4 times/day	76.0 (57)	85.3 (180)	82.9 (237)
p ~^b^		0.097	
Total	100.0 (75)	100.0 (211)	100.0 (286)

a *High: University or vocational school; low: high school, middle school, primary school, or less*.

b *From the chi-squared test for heterogeneity*.

c *Manufacturing worker, storage worker, or forklift driver*.

d *Light: Sedentary or other light work; heavy: heavy or very heavy work*.

#### Workplace Conditions

The mean worksite temperature was 4°C (−2°C in cold storage, 6°C in manufacturing halls, and 21°C in offices), but the temperature varied from −22 to 23°C. The mean temperature was 10°C for highly-educated workers and 1°C for less-educated workers (*p* <0.001). In offices, the mean temperature was similar for highly- and less-educated workers (22 and 23°C, respectively), as were the respective temperatures (1 and 3°C) for those working in other settings. Overall, 65% of the workers worked in the cold (cold storage, 87%; manufacturing halls, 54%; offices, 0%), while 44% of highly-educated workers and 73% of less-educated workers worked in cold settings. The workers spent an average of 2.0 h per day at temperatures below 0°C, with no difference between the high- and low-educated workers (1.8 vs. 2.1 h/day; *p* ~ 0.317). Overall, 83% of the workers moved between cold and warmer sites at least four times a day with little difference between the educational classes (Table 1).

Relative humidity averaged 47% (SD, 13%; range 27–72%), with higher humidity for more (52%) than less (45%) educated workers (*p* <0.001). For the more and less-educated workers in office settings, the mean relative humidity was similar (64 and 63%, respectively), as was the relative humidity for those who worked elsewhere (46 and 45%, respectively).

The distribution of air velocity was skewed, with a median of 0.38 m/s (range, 0.01–3.00 m/s). The median air velocity was exceeded for 67% of the highly-educated group vs. 44% of the less-educated group (*p* = 0.001). Among the office workers, the median air velocity was 0.44 m/s independent of education; in non-office workers, it was higher in the educated vs. non-educated group (0.40 and 0.34 m/s, respectively).

Overall, 98% of the workers reported air conditioning at the worksite.

#### Subjective Assessment of Cold

The temperature at which the respondents reported being “cold” had a right-skewed distribution (median, 15°C). For highly-educated workers, the median subjective cold temperature was 20°C, with no difference for those working in offices or elsewhere. For the less-educated workers, the median cold temperature was 15°C, but the medians differed for those working in offices vs. elsewhere (10 and 15°C, respectively).

Temperatures > 15°C were considered as cold by 57% of the highly-educated workers vs. 45% of the less-educated workers (*p* ~ 0.161), whereas 29 and 14%, respectively, considered as temperatures > 20°C cold (*p* ~ 0.006).

#### Thermal Insulation of Clothing

The basic thermal insulation of clothing averaged 1.23 clo (SD, 0.26 clo; range, 0.35–2.21 clo); it was lower in highly- (1.09 clo) vs. less (1.28 clo) educated workers (*p* <0.001) and lower in office staff (0.81 clo) than in workers in other job categories (1.28 clo) (*p* <0.001). A two-way breakdown of the clo means according to education and worksite temperature (in classes: high education and warm, high education and cold, low education and warm, and low education and cold) showed mean clo values of 0.94, 1.28, 1.33, and 1.26, respectively (*p* <0.001). Therefore, only those highly-educated workers who worked at ≥ 10°C had low clo values, while all the participants who worked at <10°C had relatively high thermal insulation levels.

### Work Ability

**Figure 2:**
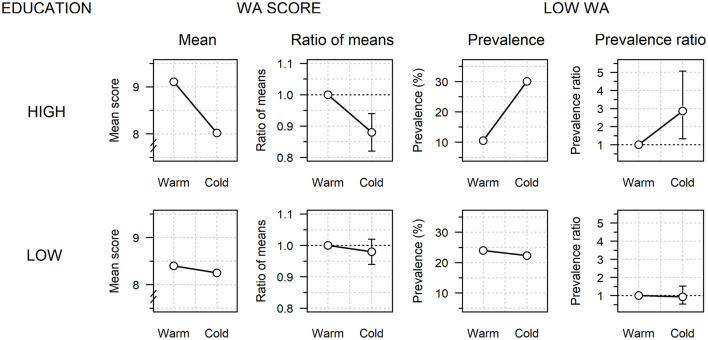
Perceived work ability (WA) according to worksite temperature (warm, 10–23°C; cold, −22–10°C) among highly- and less-educated workers. Mean WA score and prevalence of a low WA score (<8) are based on a temperature × education interaction model after adjustment for sex, age, job category, physical work strain, moving between cold and warm sites, thermal insulation of clothing, relative humidity, and air velocity. The cold vs. warm ratios of the model-adjusted means and adjusted prevalence ratios are also shown (vertical bars: 95% CIs).

Figure 2 summarizes how WA is associated with worksite temperature, how it varies by educational class, and how the WA–temperature association differs between educational classes. Table 2 provides more details. The crude overall mean WA score was 8.32 (SD, 1.33; range, 4–10) and it was slightly lower among highly- vs. less-educated workers (8.20 vs. 8.35, respectively; *p* ~ 0.482). The crude mean WA was 2% (95% CI, 0–5%) higher for workers at cold worksites than for those at warm sites. However, the trends differed by education, with a marginal decline of 2% (95% CI, −8 to 4%) in the cold among highly-educated workers, but an increase of 4% (95% CI, 1–7%) among the less-educated workers. According to the unadjusted analysis, the cold-related decline of WA in the highly-educated group was 6% (95% CI, 0–12%) greater than the change in the low-educated group.

**Table 2:** Crude and model-based adjusted^a^ work ability (WA) score and prevalence of low WA (<8) according to worksite temperature and education.

**Work ability measure**	**Education**	**Temperature**			**Cold vs. warm ratio of means/ prevalence ratio**	**Ratio of cold-warm ratios between high-and low-educated workers**
		**All [−22 to 23^**°**^C]**	**Warm [10 to 23^**°**^C]**	**Cold [−22 to 10^**°**^C]**		
**Crude**						
Mean WA score	All	8.32	8.18	8.37	1.02 (1.00 to 1.05)	
	High	8.20	8.31	8.13	0.98 (0.92 to 1.04)	0.94 (0.88, 1.00)
	Low	8.35	8.13	8.43	1.04 (1.01 to 1.07)	1
Prevalence of low WA (%)	All	23.0	22.2	23.3	1.05 (0.77 to 1.39)	
	High	25.1	18.4	29.1	1.58 (0.81 to 2.66)	1.70 (0.87, 2.86)
	Low	22.4	23.6	22.0	0.93 (0.66 to 1.28)	1
**Adjusted**						
Mean WA score	All	8.30	8.55	8.20	0.96 (0.92 to 1.00)	
	High	8.33	9.11	8.02	0.88 (0.82 to 0.94)	0.90 (0.84, 0.96)
	Low	8.29	8.40	8.25	0.98 (0.94 to 1.02)	1
Prevalence of low WA (%)	All	22.8	20.5	23.8	1.16 (0.69 to 1.82)	
	High	23.0	10.5	30.1	2.87 (1.33 to 5.07)	3.09 (1.43, 5.45)
	Low	22.8	24.0	22.3	0.93 (0.53 to 1.52)	1

a *Adjusted for sex, age, job category, physical strain at work, relative humidity, air velocity, moving between cold and warm sites, thermal insulation of clothing (clo), interaction temperature × education, and stratified sampling*.

Once allowance was made for the eight adjustment factors and the interaction between cold and education, the pattern was even clearer, although the overall effect of cold on WA reversed from an increase of 2% to a decrease of 4% (95% CI, 0–8%). In the adjusted analysis, the WA score decreased in the cold by 12% (95% CI, 6–18%) among the highly-educated workers vs. only 2% (95% CI, −6 to 2%) among the less-educated workers, which implied a 10% (95% CI, 4–16%) greater decline in WA score in the cold among the highly-educated workers. The above analyses were repeated after excluding office workers because 63% of the highly educated staff worked outside offices and stayed at <10°C (87% in cold storage and 54% in manufacturing halls). In this subsample including only non-office workers, the mean WA score also declined in the cold by 10% more (95% CI, 0–16%) in the highly-educated workers vs. the less-educated workers.

A more specific comparison of university-educated workers alone (*n* = 37) vs. all the others (*n* = 249) resulted in a 14% (95% CI, 6–23%) greater cold-related decline in mean WA score among the former.

For 22.8% of the participants, the WA score was classified as low (<8), with a slightly higher crude prevalence of low WA among highly- vs. less-educated workers (25.1 vs. 22.4%, respectively; *p* ~ 0.709). The overall crude prevalence of a low WA showed no significant differences between workers at the cold and warm sites. However, the highly-educated workers alone showed relatively high prevalence ratios for a low WA at cold worksites (crude: 1.58; 95% CI, 0.81–2.66; adjusted: 2.87; 95% CI, 1.33–5.07). The prevalence of a low WA increased in the cold by 3.09 times (95% CI, 1.43–5.45) more among highly- vs. less-educated workers. This ratio increased to 8.59 (95% CI, 2.69–15.46) when university-educated workers were compared with all the other workers. In the subsample of non-office workers, the prevalence of a low WA increased 3.11 times more (95% CI, 1.47–5.50) in the cold among the highly-educated workers.

## Discussion

### Summary of Findings

This study revealed that highly-educated chicken industry workers who worked at −22 to 10°C had a worse perceived WA than those who worked at 10–23°C, while less-educated workers showed similar adjusted WA values at both the temperatures. Cold-related worsening of WA among educated individuals was even greater when a stricter definition of high education was used. The finding was adjusted for relevant confounding factors and it remained unchanged after the exclusion of office staff, who mainly consisted of educated workers who worked at higher temperatures than the others. Therefore, we interpret our findings in terms of some undefined characteristics related to a higher education level that strengthens the effect of cold on WA. This finding is directionally similar to that in our previous study, which showed a large and consistent increase in performance degradation from lower to higher educational classes which, according to the judgment of the worker, was caused by workplace cold (1). This study used actual measured temperatures at the workplace and suggested a greater impairment of WA due to cold among highly- vs. less-educated workers.

### Education

Educational level is a strong determinant of a wide range of health outcomes in the general population, but its significance in occupational settings has remained less examined. Educational level is part of a complex of factors comprising attained education, social class, income, and occupational position, the separate effects of which are not easily identifiable. Highly-educated people have better subjective health (11), better work and functional abilities (20), and a longer life expectancy (by 1–4 years) (21) than less-educated people. In this complex of factors, attained education is of primary importance since it usually remains unchanged throughout one's adult life, is easily measured, and is strongly associated with health. The reasons underlying the wide educational differences in health are not well-understood, but they likely result in a combined effect of a number of material, behavioral, and lifestyle factors such as income, smoking, alcohol consumption, and diet (22). Education is of particular significance because it produces knowledge and skills and enhances one's cognitive abilities (23).

### Previous Studies

Studies relating education to WA in various industries have observed better WA among highly- vs. less-educated workers. A Finnish study of municipal workers noted a positive association between WA and university-level education (24); this finding is similar to that of studies of food supply factory workers (25) and petrochemical industry workers in Iran (26), while a low education level was associated with a poor WA among Brazilian public health institution workers (27). Mazloumi et al. (26) explained educational differences in WA based on the better job skills of educated workers. Only one previous study reported an association between workplace cold and poor self-assessed WA, but it failed to consider how educational levels of the workers might interfere with this association (15).

### Reasons for the Worse WA Among Highly-Educated Workers

The effect of cold on WA was estimated to be greater among highly-educated workers despite them working at warmer temperatures (10°C) than less-educated workers (1°C), fewer of them (44.0 vs. 72.5%) working in the cold, and them having a higher BMI (25.1 vs. 23.7 kg/m^2^), which is protective against cold (28). However, highly-educated workers were less likely to perform heavy work (40 vs. 64%), they had higher air velocity at their worksites (66.7 vs. 44.1% m/s), and had lower clo values than their less educated counterparts (1.09 vs. 1.28), all of which should contribute to a higher occurrence of body cooling and related adverse effects. Highly-educated workers also spent 1.8 h/day at <0°C and ~three quarters of them moved between warm and cold working sites at least four times a day, which may have caused thermal stress (29). In fact, highly-educated workers considered a higher temperature (20°C) as “cold” than their less-educated counterparts (15°C) and a normal office temperature of 20°C was considered “cold” by 29 and 14% of the workers, respectively. The results were adjusted for most of the factors mentioned above, but the possibility of residual confounding exists due to the way the adjustment variables were classified.

Considering that, with declining ambient temperature, heat production to maintain body temperature in a lightly clothed individual starts to increase at 22–27°C (30, 31) and the recommended indoor temperature in Thailand is 26°C (32); cold symptoms and consequent reports of a poor WA are likely among highly-educated workers who work at approximately 10°C. This temperature is also considered cold by international standards (17). The low thermal insulation of clothing can partly explain the findings, especially in the 37% of the highly-educated staff who worked in offices and whose thermal insulation of clothing was only 0.81 clo. Especially, in offices, effective air conditioning can produce symptoms if thermal insulation is inadequate (33). It is possible that some office workers functioning at around 20°C may not recognize office conditions as being cold enough to necessitate heavier clothing. However, workers with high levels of education working in cold storages or manufacturing halls (63%) who had higher thermal insulation (1.26–1.33 clo) often reported a poor WA. Our findings could be partly interpreted as educated and wealthier people having heightened awareness of health issues, increased perception of their personal vulnerability, and greater willingness to report symptoms (34). Nevertheless, the WA score used is a valid predictor of long-term morbidity (13); therefore, we believe that our findings depict a true association between workplace cold, WA, and education.

### Strengths and Limitations

The primary strength of this study is that we addressed the potential modifying effect of education on how workplace cold is associated with WA. Because education is a strong determinant of health, it could also modify the exposure-outcome relations, as it actually did in this study. This study had several limitations. First, the non-probabilistic sampling scheme, which was dictated by practical reasons, may have caused bias. However, marked distortions in educational differences in WA were unlikely because we controlled for relevant confounders. Second, the ambient temperature measured at the regular working area of the worker may not accurately reflect the actual cold exposure of each worker. Third, uncontrolled ergonomic factors may have been mixed with the effects of cold. Fourth, in this observational study setting, the independent effect of protective clothing could have been mixed with other factors, such as job category, making it difficult to identify. Due to the study population and the questions used, care should be exercised when generalizing our results. In addition, a health-based selection of highly- vs. less-educated workers to various job categories and more vs. less physically demanding tasks could not be controlled. Finally, the low number of workers with diagnosed diseases prevented us from controlling for preexisting medical conditions.

### Conclusion

The cold-related disadvantage of highly-educated workers is likely to be reflected in increased long-term morbidity and sickness-related absences. All the workers, educated workers in particular, should be given intensified health education to increase the awareness of cold hazards; they should be reminded to wear thicker clothing and more clothing layers that effectively reduce cold harm (2), air conditioning should be set appropriately (33); and workers should be advised to avoid unnecessary stays in cold and consume hot beverages to maintain proper hydration. In addition, the current practices of cold protection should be critically reviewed considering this particular group of workers. There is a need to establish regulatory standards for cold workplaces that do not currently exist in Thailand. We also suggest more specific studies to clarify the reasons for cold sensitivity in this subgroup of workers. This information becomes increasingly relevant considering that global warming affects this area the most (35), increasing the contrast between outdoor heat and workplace cold.

## Data Availability Statement

The data are confidential. Requests to access the datasets should be directed to WP, phwpp2@gmail.com.

## Ethics Statement

The studies involving human participants were reviewed and approved by Ethical Review Committee for Human Research, Faculty of Public Health, Mahidol University, Bangkok, Thailand. The patients/participants provided their written informed consent to participate in this study.

## Author Contributions

WP and SN: conceptualization and methodology. WP, PK, WL, TI, JJ, and SN: validation and writing – original draft preparation. SN: formal analysis. WP and PK: investigation. WP, PK, WL, CT, TI, JJ, and SN: writing – review and editing. All authors have read and agreed to the published version of the manuscript.

## Funding

This work was supported by Mahidol University, Bangkok, Thailand.

## Conflict of Interest

The authors declare that the research was conducted in the absence of any commercial or financial relationships that could be construed as a potential conflict of interest.

## Publisher's Note

All claims expressed in this article are solely those of the authors and do not necessarily represent those of their affiliated organizations, or those of the publisher, the editors and the reviewers. Any product that may be evaluated in this article, or claim that may be made by its manufacturer, is not guaranteed or endorsed by the publisher.
